# The Impact of BWM‐T Versus Brief Mindfulness Induction on Stress, Anxiety and Self‐Efficacy in University Students: Immediate and Short‐Term Outcomes of RCT on Psychological Well‐Being and Autonomic Balance

**DOI:** 10.1002/brb3.71471

**Published:** 2026-05-08

**Authors:** Mauro Cozzolino, Marco Borgese, Giovanna Celia

**Affiliations:** ^1^ Department of Human, Philosophical and Educational Sciences University of Salerno Fisciano Italy; ^2^ Department of Human Theory and Applied Sciences Univesity ECampus Novedrate Italy

**Keywords:** anxiety, autonomic balance, brain waves modulation technique, heart rate variability, mind–body interventions, mindfulness, stress

## Abstract

**Background**: University students face increasing levels of stress, anxiety, and self‐efficacy, which may disrupt autonomic balance. While many mind–body interventions require sustained practice, growing evidence suggests that even a single session can yield immediate benefits. This randomized controlled trial compared the immediate and short‐term effects of the brain wave modulation technique (BWM‐T) and a brief mindfulness induction on psychological and physiological outcomes in university students.

**Methods**: 68 students were randomly assigned to either BWM‐T or a brief mindfulness induction. State anxiety (STAI‐Y1), psychological distress (distress thermometer), generalized self‐efficacy scale (GSES), and 5 min heart rate variability (HRV) indices were assessed at baseline (T0), immediately after the session (T1), and 4 h later (T2). Mixed‐design ANOVAs with Time (T0, T1, T2) as a within‐subject factor and group (BWM‐T vs. brief mindfulness induction) as a between‐subject factor were conducted, followed by paired‐sample *t*‐tests where appropriate.

**Results**: Across the total sample, significant main effects of Time emerged for state anxiety, distress, and self‐efficacy (ps < 0.001), indicating reductions in anxiety and distress and increases in self‐efficacy from pre‐to post‐session that were largely maintained at the 4 h follow‐up. No significant time × group interactions were found for psychological outcomes, suggesting comparable trajectories for BWM‐T and mindfulness. For HRV indices, a significant time × group interaction was observed only for VLF power. VLF decreased from T0–T1 in the BWM‐T group, whereas no systematic changes were detected in the mindfulness group. All other HRV parameters (SDNN, rMSSD, pNN50, LF, HF, LF/HF) remained stable over time, with no differential group effects.

**Conclusions**: A single 16 min session of either BWM‐T or a brief mindfulness induction yielded rapid improvements in self‐reported anxiety, distress and GSES in university students, with effects persisting up to 4 h. Physiological effects were limited to a short‐lived reduction in VLF power following BWM‐T, while other indices of vagally mediated HRV did not change significantly. These findings support the use of brief mind–body practices as feasible tools for acute stress regulation in academic settings, while underscoring the need for larger, adequately powered trials and more comprehensive autonomic assessments.

## Graphical abstract

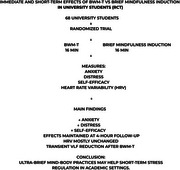

## 1 Introduction

In recent years, research has shown a rise in distress and anxiety in university students (Oswalt et al. 2020). Analyzing the various causes, several factors have emerged as central, including competitive academic environments, increased workload, difficulties in time management, and uncertainty about future prospects (Yotsidi et al. 2023). One aspect that highlights the impact of these stressors is that they not only reduce students' subjective well‐being but also compromise essential academic functions. These include working memory, attention, and executive functions in general (Pascoe et al. 2020). Elevated distress has also been associated with poorer sleep quality, depressive symptoms, and increased risk of academic dropout (Abid et al. 2021).

When it comes to university students, the concept of self‐efficacy, defined as a student's belief in their ability to organize and execute the actions necessary to achieve academic goals (Bandura 1997), emerges as an essential mental resource. Several studies have shown that higher levels of self‐efficacy are related to lower levels of academic distress, anxiety (Zajacova et al. 2005; Chemers et al. 2001), and overall improved academic performance and perseverance (Usher et al. 2019). Furthermore, longitudinal studies have shown that students with higher self‐efficacy tend to display greater resilience when faced with academic demands and greater adaptive coping techniques (Talsma et al. 2020).

Although self‐efficacy is often described as relatively stable, Bandura (1997) emphasizes that it is a context‐dependent belief that can shift rapidly in response to changes in emotional arousal and perceived control. When interventions reduce anxiety and physiological activation, individuals momentarily reinterpret their abilities as more effective. This is consistent with stress‐appraisal models (Lazarus et al. 1984) and with psychophysiological frameworks showing that improvements in autonomic regulation support a greater sense of control (Thayer et al. 2012). For these reasons, self‐efficacy can show short‐term, state‐like fluctuations, making it an appropriate proximal outcome in brief stress‐reduction interventions.

Stress responses involve not only psychological reactions but also rapid and dynamic physiological adjustments. Contemporary psychophysiological models emphasize the role of the autonomic nervous system (ANS) in stress appraisal and regulation, particularly the balance between sympathetic activation and parasympathetic regulation (Thayer et al. 2012). Stress‐related reductions in parasympathetic activity have been associated with increased heart rate, heightened muscle tension, gastrointestinal discomfort, and cardiovascular dysregulation (Kim et al. 2019; Chu et al. 2025).

Given its central role in autonomic regulation, one of the most widely used physiological markers of stress‐related autonomic flexibility is heart rate variability (HRV). HRV refers to the fluctuation in the time intervals between consecutive heartbeats, reflecting the dynamic interplay between sympathetic and parasympathetic branches of the autonomic nervous system (Shaffer et al. 2017). Higher vagally‐mediated HRV indexes greater autonomic flexibility and self‐regulatory capacity. HRV has emerged as a reliable measure of autonomic nervous system function and reflects the body's ability to manage stress (Shaffer et al. 2017; Laborde et al. 2017), with higher values generally indicating better autonomic balance (Shaffer et al. 2017).

A growing body of studies has shown that it is possible to intervene to rebalance HRV using techniques that connect the mind and body. These include, for example, slow‐paced breathing, meditation, biofeedback, and yoga (Chen et al. 2021). The effects produced by these types of interventions can acutely modulate HRV and improve stress‐related outcomes (Wielgosz et al. 2019). However, some limitations have emerged in the use of this type of intervention, among the main ones being the need for qualified professionals to learn the techniques and a significant amount of time before producing stable effects (Dobkin et al. 2012).

These constraints have generated growing interest in interventions that can elicit rapid autonomic and psychological shifts with minimal training demands. In this context, a recent line of research has focused on brief, single‐session practices capable of producing measurable psychophysiological effects (Wu et al. 2021; Wen et al. 2022).

Brief applications of mindfulness induction, slow‐paced breathing, or HRV biofeedback have been shown to reduce acute distress, enhance perceived control. These practices can also increase parasympathetic activity and HRV in the short term (Tang et al. 2007; Toan et al. 2024). For instance, brief applications of slow‐paced breathing or HRV biofeedback have been shown to rapidly enhance vagal tone and increase parasympathetic activity (Lehrer et al. 2014), resulting in a short‐term rise in HRV, which is commonly regarded as a marker of physiological flexibility and self‐regulatory capacity (Shaffer et al. 2017).

These effects can be interpreted through models of regulatory preactivation, which propose that contemplative and breathing‐based practices can transiently activate autonomic homeostatic mechanisms, thereby stabilizing physiological state and reducing reactivity to subsequent demands (Tang et al. 2009; Thayer et al. 2009). Complementary perspectives, including Polyvagal Theory (Porges 2007) and the Neurovisceral Integration Model, similarly highlight the links between vagal functioning and executive control (Smith et al. 2017). Collectively, these frameworks offer a compelling rationale for studying brief mind–body practices capable of inducing immediate and short‐lasting improvements in autonomic balance and psychological well‐being.

Within this framework, the first intervention examined was brain wave modulation technique (BWM‐T), a brief, accessible, and easily mind–body practice. Several studies have already demonstrated the effectiveness of BWM‐T and related mind–body interventions for reducing perceived distress and enhancing autonomic balance, even after a single session (Cozzolino et al. 2018; Cozzolino et al. 2020a; Cozzolino et al. 2021; Cozzolino et al. 2020b; Cozzolino et al. 2020c; Borgese et al. 2025).

The second intervention employed in this study was a brief Mindfulness induction modeled after the protocol used by Watford (2015), who demonstrated that short, audio‐guided practices incorporating focused attention and present‐moment awareness can effectively modulate emotional states and psychophysiological responses. Unlike standardized multi‐session programs such as MBSR or MBCT, the present intervention consists of a brief contemplative exercise designed to elicit short‐term regulatory effects. Consistent with previous findings, brief inductions of this type have been shown to influence state anxiety, attentional control, and autonomic markers such as HRV, even following a single session (Watford 2015; Lin et al. 2019; Tang et al. 2015; Toan et al. 2024).

Although both BWM‐T and brief mindfulness induction fall under the umbrella of mind–body practices, they may engage partially distinct regulatory pathways. BWM‐T could be conceptualized as a predominantly bottom‐up oriented intervention insofar as it stabilizes regulation primarily through embodied attentional anchoring and interoceptive discrimination (Cozzolino et al. 2018). In this framework, autonomic modulation is not a random byproduct but the downstream expression of an attentional–interoceptive process through which individuals recognize their internal state and adjust it accordingly (Theadom et al. 2015).

This mechanism is consistent with models emphasizing the role of bodily states in autonomic regulation, such as the polyvagal theory (Porges 2007) and the neurovisceral integration framework (Thayer et al. 2012; Smith et al. 2017), both of which highlight how vagally mediated pathways and feedback from the body contribute to adaptive stress responses.

In contrast, brief mindfulness induction operates mainly through top‐down cognitive regulation processes, attentional control, present‐moment monitoring, and reappraisal, functions strongly associated with prefrontal and anterior cingulate networks implicated in executive regulation and autonomic modulation (Hölzel et al. 2011; Tang et al. 2015). These top‐down processes can indirectly influence autonomic balance by altering cognitive load, appraisal, and attentional stability.

This framing clarifies the nature of the intervention and distinguishes it from multi‐week mindfulness programs requiring extended practice or instructor certification. However, the duration and logistical demands of traditional MBI programs, often requiring eight weeks of practice, daily home assignments, and trained facilitators, can present significant barriers for students navigating rigorous academic schedules (Khoury et al. 2013; Britton 2019).

Recent literature has exploited this limitation by exploring the impact of ultra‐short meditation formats, raising the possibility that even a single session can produce effects on both physiological indicators such as HRV and psychological indicators such as distress perception and attention control (Lin et al. 2019; Toan et al. 2024; Tang et al. 2015). Despite promising preliminary findings, there is limited evidence on whether the effects of single‐session interventions persist beyond the immediate post‐intervention window, particularly within the first few hours, a timeframe especially relevant for real‐world academic settings where stressors may fluctuate throughout the day (Short et al. 2015; Howarth et al. 2019; Chojnacka et al. 2016; Beiter et al. 2015).

Psychological interventions aimed at improving stress regulation and well‐being in university students have been widely investigated. Mindfulness‐based and cognitive‐behavioral approaches have demonstrated reductions in anxiety and distress in academic populations (Regehr et al. 2013; Conley et al. 2015; Tinella et al. 2025). However, most studies have examined multi‐session formats, and comparative evidence on ultra‐brief single‐session interventions remains limited.

The choice of a 4 h follow‐up in the present study was based on both conceptual and methodological considerations. From a psychophysiological standpoint, short‐term regulatory changes in autonomic activity and emotional arousal naturally fluctuate across the day as individuals resume routine academic or daily activities, which can reintroduce cognitive load and stress‐related demands (Thayer et al. 2012; Smith et al. 2017). A 4 h window represents a critical ecological timeframe in university settings, during which students typically encounter new stressors, such as classes, deadlines, interpersonal interactions, or performance‐related evaluations, known to modulate affective and autonomic states (Pascoe et al. 2020; Beiter et al. 2015). Assessing this specific window therefore allows for evaluating whether the immediate benefits of BWM‐T or a brief mindfulness induction extend into a period in which distress and physiological challenges naturally re‐emerge.

Furthermore, recent work on ultra‐brief contemplative and breath‐based interventions has shown that improvements in mood, attentional regulation, and autonomic recovery may persist for 1–2 h after a single session (Kirk et al. 2020; Streeter et al. 2012; Toan et al. 2024), but the stability of these effects beyond this timeframe remains unclear. By adopting a 4 h interval, long enough to capture meaningful physiological fluctuations but short enough to remain within the window of expected proximal intervention effects, the present study aimed to address this gap and provide a more ecologically valid understanding of how single‐session mind–body interventions may support stress regulation during the unfolding of a typical academic day.

Building on this gap, the current study investigates whether the immediate benefits of brief mind–body interventions extend into the subsequent hours. Specifically, we compare the short‐term effects of a single session of BWM‐T and a brief mindfulness induction on psychological outcomes state anxiety, acute distress, and general self‐efficacy, with physiological outcomes HRV indices, in university students, with follow‐up assessments conducted 4 h post‐intervention. The primary research question concerns the extent to which both interventions produce benefits that persist up to 4 h after completion and whether BWM‐T and brief mindfulness induction differ in their short‐term effects on autonomic balance.

## 2 Materials and Methods

### 2.1 Sample

An a priori power analysis was conducted for a mixed‐design ANOVA with two groups and three time points, focusing on the group × time interaction, which represents the critical effect of interest in this design. Following conventions for medium effects in repeated‐measures designs (Cohen 1988), we assumed an effect size of *f* = 0.25, α = 0.05, and power (1–β) = 0.80. Under these conditions, methodological guidelines for mixed ANOVA indicate a required total sample size of approximately *N* = 68 participants (see Table 1). The final sample reached this threshold, although with no margin to account for potential deviations from statistical assumptions. The study should therefore be considered adequately powered to detect medium effects, but with limited sensitivity to smaller interaction effects.

**TABLE 1:** Sample characteristics of participants by group.

Group	N	Age, *M* (SD)	Female, *n* (%)
BWM‐T	34	27.35 (6.84)	20 (58.8)
Brief mindfulness induction	34	25.94 (5.67)	27 (79.4)
Total	68	26.65 (6.28)	47 (69.1)

*Note*: Values represent means (M) and standard deviations (SD) or number and percentage of female participants.

A total of 72 university students initially expressed interest in the study through recruitment announcements distributed via academic mailing lists and departmental websites. Inclusion criteria required participants to be at least 18 years old and free from severe physical or psychological conditions. No participant reported previous experience with BWM‐T or brief mindfulness‐based practices. Following eligibility screening, all 72 individuals were admitted, however four were excluded due to incomplete HRV data or missing questionnaire responses, resulting in a final sample of 68 participants.

The sample exhibited heterogeneous age characteristics, with a mean age of 27.5 years (SD = 13.48). This distribution reflects the typical demographic profile of Italian university programs that include both traditional students and individuals returning to education later in life. The final sample consisted of 47 females (69.1%) and 21 males (30.9%), consistent with gender distributions in the participating academic departments.

Each participant filled out a series of psychological questionnaires and had their HRV measured at three different times: before the intervention (T0), right after the intervention (T1), and 4 h later (T2). Each participant was given a unique ID to track their data while keeping their identity private. The informed consent document explained that participants could leave the study at any time without facing any negative effects.

All the information gathered was kept anonymous and stored in a coded way to protect personal details and follow data security rules. The methods used in this research followed the ethical guidelines set by the Italian Association of Psychology (AIP) and matched the ethical standards from the 1964 Helsinki Declaration and its later updates. The study plan was examined and accepted by the Ethics Committee at the University of Salerno on June 11, 2024, with the reference number 0186309.

### 2.2 Measures

All measures were digitized and made available to the participants via a shared computer link through an online platform.

#### 2.2.1 Distress Thermometer

Perceived short‐term stress was assessed using the distress thermometer (DT), a single‐item screening tool developed by Jacobsen et al. (2005). This tool is recognized for its high sensitivity and specificity in measuring short‐term stress, as noted in Snowden et al. (2011). The DT has been previously validated for use in the Italian context, as demonstrated by Cozzolino et al. (2020a). Participants were asked to rate their current level of stress on an 11‐point scale, ranging from 0 (indicating no distress) to 10 (representing maximum distress), as described by Jacobsen et al. (2005). Due to its concise and single‐question format, the DT is especially appropriate for use in stress management studies involving university students.

#### 2.2.2 State‐Trait Anxiety Inventory Form Y

Anxiety levels were assessed using the state–trait anxiety inventory form Y (STAI‐Y) (Spielberger et al. 1983), a self‐report questionnaire designed to measure both current (state) anxiety and a general predisposition toward anxiety (trait). This tool has shown strong psychometric support in studies conducted with Italian populations (Pedrabissi et al. 1989). In this research, only the state anxiety subscale (S‐ANX) was used, consisting of 20 items. Each item is rated on a 4‐point Likert scale, ranging from 1 (“not at all”) to 4 (“very much so”), resulting in a total score that ranges from 20 (indicating low anxiety) to 80 (indicating high anxiety). Higher scores on this scale are associated with increased anxiety linked to the participant's current emotional condition.

#### 2.2.3 Generalized Self‐Efficacy Scale

The generalized self‐efficacy scaleGSES (Sibilia et al. 1995) was used to assess perceived efficacy in managing challenges and regulating one's responses to situational demands. The scale consists of 10 items rated on a 4‐point Likert scale (1 = not at all true; 4 = exactly true), with a reliability coefficient of α = 0.86.

Although often conceptualized as a relatively stable belief, self‐efficacy can also show short‐term, state‐like fluctuations in response to changes in emotional arousal and perceived control, as suggested by Bandura's theory of efficacy beliefs and by stress‐appraisal models (Bandura 1997; Lazarus et al. 1984). For this reason, the GSES has been employed in previous studies to capture proximal changes in perceived coping ability following brief stress‐regulation interventions. In the present study, the GSES was therefore used as a concise and validated measure of students’ perceived capacity to manage immediate academic and emotional demands. The implications of this choice are further addressed in the Discussion.

#### 2.2.4 Heart Rate Variability

HRV was recorded using a Polar H10 chest strap, which provides R–R interval measurements with high temporal resolution (1 ms) and has been validated against standard electrocardiogram (ECG) for short‐term HRV analysis (Gilgen‐Ammann et al. 2019; Gamelin et al. 2007). Participants were seated comfortably, asked to breathe spontaneously and instructed to minimize movement during the recording. All assessments were conducted in a quiet room with stable temperature and controlled lighting. Recordings lasted 5 min, consistent with the guideline minimum for short‐term HRV acquisition (Task Force, 1996; Shaffer et al. 2017). Two recordings were collected, a baseline assessment before the intervention (T0) and a second assessment immediately after the intervention (T1). A third HRV measurement was performed four hours later (T2).

R–R interval data were exported as .txt files and processed in Kubios HRV Premium (version 4.2.0). Preprocessing followed the software's standard pipeline, including artifact correction with Kubios automatic beat correction (threshold‐based algorithm), detrending using the smoothness priors method (λ = 500), interpolation, and resampling at 4 Hz as recommended for spectral analysis. HRV indices were calculated in the frequency domain using Fast Fourier Transform (Welch's periodogram). HRV was derived from interbeat interval recordings and analyzed using Kubios HRV software (version 4.2.0). Time‐ and frequency‐domain indices were computed according to standard procedures.

Respiratory rate (RP) was estimated from the interbeat interval time series using Kubios HRV software. This estimate reflects breathing frequency (expressed in Hz) derived from cardiorespiratory oscillations, but does not provide information on respiratory depth. In line with recent methodological recommendations, this measure was used to characterize condition‐related changes in breathing frequency and to contextualize HF‐HRV findings, rather than as a direct measure of respiratory physiology (Quigley et al. 2024).

### 2.3 Design

This study employed a parallel‐group randomized controlled trial with one between‐subject factor (BWM‐T vs. brief mindfulness induction) and one within‐subject factor (time, baseline, immediately after the intervention, and 4 h later). Before participation, all students received written information describing the study aims, procedures, potential risks, and benefits, and their right to withdraw at any time without consequences. Written informed consent was obtained from each participant.

Randomization was performed using research randomizer (Urbaniak et al. 2013), which generated a blocked randomization sequence (block size = 4) to ensure balanced group allocation with a 1:1 ratio. Group assignment was concealed using sequentially numbered entries accessible only after participants completed the baseline assessment. Due to the nature of the interventions, participants could not be blinded to group assignment, however, data processing, and HRV extraction were performed by an assessor blinded to the intervention condition.

A total of 68 participants were randomized, with 34 assigned to the BWM‐T group and 34 to the brief mindfulness induction group. No significant differences between groups were observed at baseline on demographic or psychological variables, supporting successful randomization. In the frequency domain, HF (0.15–0.40 Hz), LF (0.04–0.15 Hz), and VLF (<0.04 Hz) components were included. The LF/HF ratio was reported but interpreted cautiously due to well‐documented methodological concerns (Billman 2013). Natural logarithmic transformations were applied to HF, LF, and VLF values prior to analysis. In the time domain, HF, SDNN, rMSSD, and pNN50 were also computed (Laborde et al. 2017; Shaffer and Ginsberg 2017). RP was estimated from the cardiac signal and considered when interpreting HF changes.

### 2.4 Intervention

All participants attended a single experimental session conducted in a quiet university laboratory. Upon arrival, a standardized script was read aloud to ensure uniform instructions across conditions. Participants first completed the baseline psychological questionnaires (STAI‐Y state, DT, and GSES).

HRV was then assessed using a Polar H10 chest strap, which recorded R–R intervals for 5 min while participants sat comfortably with their eyes closed and were instructed to breathe spontaneously, and minimize movement. Environmental conditions, light, temperature, and noise level, were kept constant across all assessments.

Following the baseline recording, participants completed their assigned intervention.

Participants in the BWM‐T condition received a 16 min session delivered by a trained clinical psychologist following the standardized four‐step protocol described in previous studies (Cozzolino et al. 2020a; Cozzolino et al. 2020b). Each position was maintained for approximately 4 min, with live verbal guidance encouraging bodily awareness and stillness. The facilitator demonstrated and guided the procedure in real time to ensure adherence.

Participants in the brief mindfulness‐induction condition completed a 16 min audio‐guided contemplative exercise modeled after Watford (2015). The induction included periods of focused attention, present‐moment awareness, and silently repeated phrases. This practice represents a brief mindfulness‐related induction, rather than a full mindfulness training program. Participants were seated with their eyes closed and instructed to follow the guidance attentively.

Immediately after the intervention, a second 5 min HRV recording was obtained under the same standardized conditions as the baseline. Participants then completed the same psychological questionnaires (STAI‐Y state, DT, GSES).

Participants were instructed to refrain from consuming caffeine, alcohol, or tobacco, and to avoid strenuous physical activity or stressful tasks during the 4 h interval between the two assessments. The same restrictions were also provided prior to the baseline session. Adherence was not monitored through direct measurement but was communicated as part of the standard procedure for short‐term psychophysiological studies. At the 4 h follow‐up (T2), participants returned to the laboratory, completed the psychological questionnaires for the third time, and underwent a final 5 min HRV recording using the same procedures and equipment as in previous assessments.

### 2.5 Statistical Analysis

Before the main analyses, the dataset was inspected for missing values and outliers using boxplots. No missing data were identified. Normality, homogeneity of variance and sphericity were evaluated through Shapiro–Wilk tests, Levene's tests, and Mauchly's test, respectively. Mild deviations from normality were tolerated given the robustness of mixed‐design ANOVA, therefore, no transformations were applied.

Because participants were randomly assigned to groups and no baseline differences emerged, no covariates were included. In small randomized samples, covariate adjustment may reduce statistical power and increase model instability, thus, unadjusted models were used.

Mixed‐design ANOVAs were conducted with time (T0‐T1‐T2) as a within‐subject factor and group (BWM‐T vs. brief Mindfulness induction) as a between‐subject factor. Separate models were tested for each psychological outcome (STAI‐X1, DT, GSES) and HRV indices (HR, SDNN, rMSSD, pNN50, VLF, LF, HF, LF/HF). Estimated RP (Hz) was included to contextualize HRV changes rather than as a primary outcome. Effect sizes were reported as partial eta squared (ηp^2^), with α = 0.05.

A significant time × group interaction emerged only for VLF, F(2, 132) = 3.36, *p* = 0.038, ηp^2^ = 0.05. For this variable, within‐group paired‐sample *t*‐tests were performed to clarify the temporal pattern in each group separately. The BWM‐T group showed a significant decrease from T0–T1, whereas no significant changes were observed in the mindfulness group.

For all other variables, the time × group interaction was non‐significant; therefore, post‐hoc analyses were conducted within each group to examine temporal changes where the main effect of Time was significant. Cohen's d and 95% confidence intervals were computed for all paired comparisons.

Given the exploratory nature of the study and the relatively small sample size, no global correction for multiple comparisons was applied across all outcomes. However, when a significant omnibus effect emerged, Bonferroni‐adjusted post hoc tests were applied to the corresponding comparisons. This approach was adopted to balance the risk of Type I and Type II errors in an exploratory psychophysiological context.

## 3 Results

Results from the mixed‐design ANOVAs are reported within each subsection (3.1–3.10) for clarity. When a main effect of time or a significant time × group interaction emerged, paired‐sample t‐tests were used to clarify within‐group changes.

Descriptive statistics for the psychological variables across time points are presented in Table 2.

**TABLE 2:** Descriptive statistics of psychological and HRV outcomes for BWM‐T and brief mindfulness induction groups at pre‐ post—Follow‐up assessments (M ± SD).

Outcome	Group	Pre (T0)	Post (T1)	Follow‐up (T2)
STAI‐X1	BWM‐T	38.0 ± 9.50	33.2 ± 11.1	35.2 ± 12.5
—	Brief mindfulness induction	37.4 ± 8.87	33.6 ± 9.32	34.5 ± 9.38
DT	BWM‐T	3.35 ± 2.07	2.47 ± 1.85	2.76 ± 2.00
—	Brief mindfulness induction	3.91 ± 2.01	2.44 ± 1.83	2.59 ± 2.00
GSES	BWM‐T	29.8 ± 4.86	32.4 ± 4.25	32.0 ± 5.23
—	Brief mindfulness induction	28.4 ± 4.33	30.8 ± 4.33	30.0 ± 4.35
HR	BWM‐T	76.9 ±9.90	76.9 ± 9.35	79.8 ± 9.56
—	Brief mindfulness induction	78.6 ±13.4	77.0 ±10.5	82.0 ± 11.3
SDNN	BWM‐T	41.4 ± 15.8	41.4 ± 14.1	42.3 ± 17.7
—	Brief mindfulness induction	43.0 ± 18.0	41.8 ± 16.5	40.2 ± 16.8
rMSSD	BWM‐T	32.8 ± 13.8	34.5 ± 15.8	34.9 ± 15.4
—	Brief mindfulness induction	38.9 ± 22.8	37.8 ± 19.0	34.7 ± 16.6
pNN50	BWM‐T	16.0 ± 15.1	16.8 ± 17.5	14.8 ± 14.4
—	Brief mindfulness induction	19.1 ± 17.9	16.1 ± 16.3	15.0 ± 13.7
VLF	BWM‐T	4.04 ± 1.15	3.50 ± 1.17	3.84 ± 1.05
—	Brief mindfulness induction	3.66 ± 1.09	3.82 ± 1.26	3.56 ± 1.03
LF	BWM‐T	6.35 ± 1.02	6.31 ± 1.14	6.35 ± 1.01
—	Brief mindfulness induction	6.16 ± 1.19	6.23 ± 1.21	6.09 ± 1.07
HF	BWM‐T	6.18 ± 1.02	5.69 ± 0.935	5.81 ± 0.995
—	Brief mindfulness induction	6.10 ± 1.09	5.99 ± 1.03	5.68 ± 1.16
LF/HF	BWM‐T	1.75 ± 1.37	2.67 ± 2.24	2.15 ± 1.92
—	Brief mindfulness induction	2.11 ± 3.09	1.96 ± 1.90	2.86 ± 4.11
RP (Hz)	BWM‐T	0.24 ± 0.05	0.25 ± 0.06	0.24 ± 0.26
—	Brief mindfulness induction	0.25 ± 0.07	0.26 ± 0.06	0.26 ± 0.07

*Note*: STAI‐X1 = State‐trait anxiety inventory—state version; DT = distress thermometer; GSES = general self‐efficacy scale; HRV = heart rate variability; HR = heart rate SDNN = standard deviation of NN intervals; rMSSD = root mean square of successive differences; pNN50 = percentage of successive NN intervals > 50 ms; VLF = very low frequency power; LF = low frequency power; HF = high frequency power.; RP = estimated respiratory rate.

### 3.1 State Anxiety (STAI‐Y1)

**FIGURE 1:**
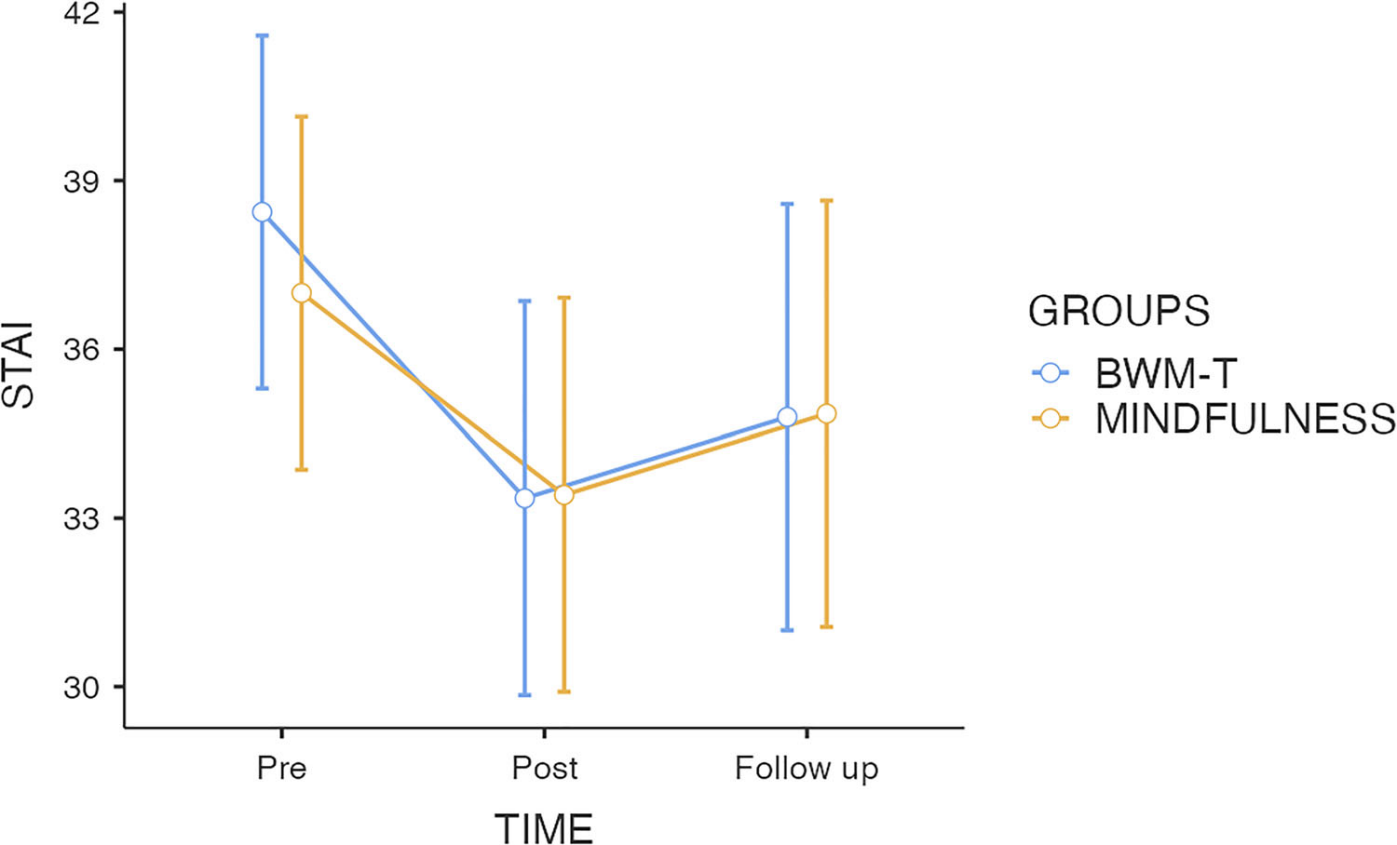
Mean state anxiety (STAI‐Y1) scores at baseline (T0), post‐intervention (T1), and 4 h follow‐up (T2) for the BWM‐T and brief Mindfulness induction groups. Error bars represent ±1 SE.

A mixed‐design ANOVA revealed a significant main effect of time on state anxiety, *F*(2, 132) = 15.88, *p* < 0.001, *ηp^2^* = 01.19, indicating a reduction across assessment points. In contrast, neither the main effect of group, *F*(1, 66) = 0.04, *p* = 0.848, *ηp^2^* = 0.00, nor the time × group interaction, *F*(2, 132) = 0.61, *p* = 0.545, *ηp^2^* = 0.01, reached significance, suggesting that both interventions produced comparable changes over time (see Figure 1).

### 3.2 Distress Thermometer (DT)

**FIGURE 2:**
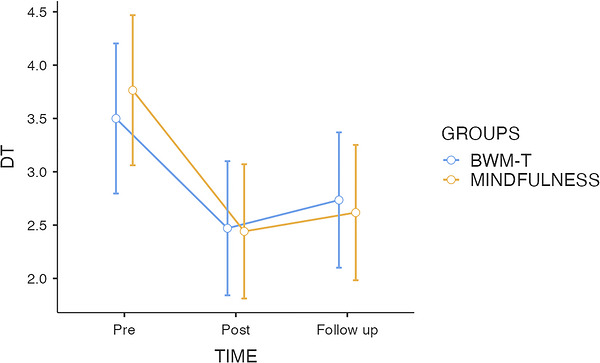
Mean psychological distress (DT) scores at baseline (T0), post‐intervention (T1), and 4 h follow‐up (T2) for the BWM‐T and brief mindfulness induction groups. Error bars represent ±1 SE.

The mixed‐design ANOVA revealed a significant main effect of time on distress levels, F(2, 132) = 20.78, p < 0.001, *ηp^2^* = 0.24, indicating a general reduction across the three assessment points. In contrast, neither the main effect of group, F(1, 66) = 0.01, p = 0.924, nor the time × group interaction, F(2, 132) = 0.53, p = 0.589, reached significance, suggesting that both interventions produced similar trajectories over time. (See Figure 2).

### 3.3 Self‐Efficacy (GSES)

**FIGURE 3:**
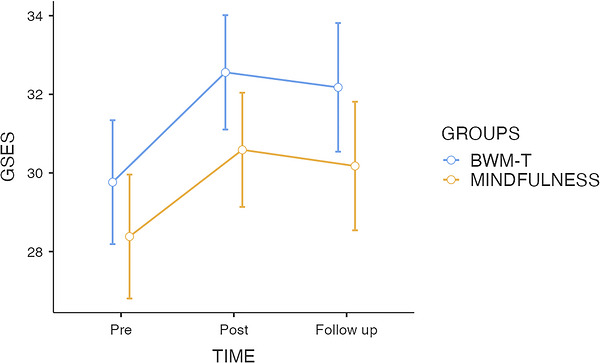
Mean general self‐efficacy (GSES) scores at baseline (T0), post‐intervention (T1), and 4 h follow‐up (T2) for the BWM‐T and brief mindfulness induction groups. Error bars represent ±1 SE.

The mixed‐design ANOVA revealed a significant main effect of time on self‐efficacy, *F*(2, 132) = 16.74, *p* < 0.001, *ηp^2^* = 0.20, indicating a general increase across assessment points. In contrast, neither the main effect of group, *F*(1, 66) = 3.43, *p* = 0.690, nor the time × group interaction, *F*(2, 132) = 0.28, *p* = 0.755, reached significance, indicating that the two interventions produced comparable trajectories over time (see Figure 3).

Descriptive statistics for HRV and physiological variables across time points are presented in Table 3.

**TABLE 3:** Results of mixed‐design ANOVAs for psychological and HRV outcomes.

Outcome	Effect	*F*(df)	*p*‐value	ηp^2^
STAI‐X1	Time	*F*(2,132) = 15.88	<0.001	0.19
—	Group	*F*(1, 66) = 0.04	0.848	0.00
—	Time × group	*F*(2, 132) = 0.61	0.545	0.01
DT	Time	*F*(2, 132) = 20.78	<0.001	0.24
—	Group	*F*(1, 66) = 0.01	0.924	0.00
—	Time × group	*F*(2, 132) = 0.53	0.589	0.01
GSES	Time	*F*(2, 132) = 16.74	<0.001	0.20
—	Group	*F*(1, 66) = 3.43	0.69	0.05
—	Time × group	*F*(2, 132) = 0.28	0.755	0.00
HR	Time	*F*(2,132) = 9.76	<0.001	0.13
—	Group	*F*(1, 66) = 0.30	0.586	00
—	Time × group	*F*(2, 132) = 0.67	0.515	0.01
SDNN	Time	*F*(2, 132) = 0.14	0.867	0.00
—	Group	*F*(1, 66) = 0.02	0.883	0.00
—	Time × group	*F*(2, 132) = 0.64	0.527	0.01
rMSSD	Time	*F*(2, 132) = 0.29	0.750	0.00
—	Group	*F*(1, 66) = 0.72	0.398	0.01
—	Time × group	*F*(2, 132) = 1.06	0.348	0.02
pNN50	Time	*F*(2, 132) = 1.29	0.279	0.02
—	Group	*F*(1, 66) = 0.09	0.765	0.00
—	Time × group	*F*(2, 132) = 0.79	0.455	0.01
VLF	Time	*F*(2, 132) = 3.36	0.038	0.05
—	Group	*F*(1, 66) = 0.94	0.393	0.01
—	Time × group	*F*(2, 132) = 0.29	0.593	0.00
LF	Time	*F*(2, 132) = 0.34	0.693	0.01
—	Group	*F*(1, 66) = 1.01	0.319	0.02
—	Time × group	*F*(2, 132) = 0.10	0.890	0.00
HF	Time	*F*(2, 132) = 0.34	0.693	0.01
—	Group	*F*(1, 66) = 0.54	0.466	0.01
—	Time × group	*F*(2, 132) = 0.10	0.890	0.00
LF/HF	Time	*F*(2, 132) = 2.15	0.121	0.03
—	Group	*F*(1, 66) = 0.09	0.772	0.00
—	Time × group	*F*(2, 132) = 0.94	0.394	0.01
RP	Time	*F*(2, 132) = 1.72	0.184	0.03
—	Group	*F*(1, 66) = 0.94	0.337	0.01
—	Time × group	*F*(2, 132) = 0.48	0.618	0.01

*Note*: ηp^2^ = partial eta squared. All tests two‐tailed.

### 3.4 HR

**FIGURE 4:**
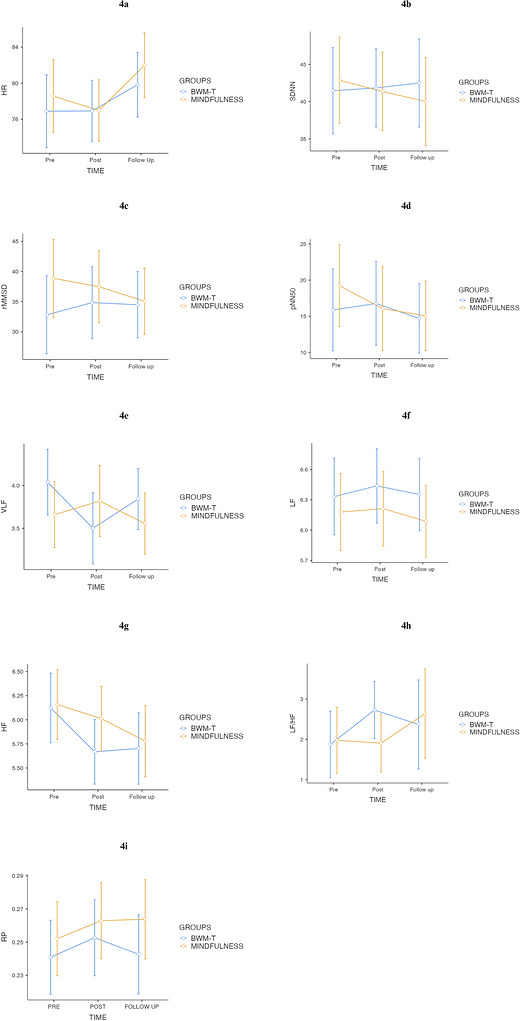
Heart rate variability (HRV) indices across time points at baseline (T0), post‐intervention (T1) and 4 h follow‐up (T2) for the BWM‐T and brief mindfulness induction groups. Error bars represent ±1 SE. (a) heart rate (HR); (b) standard deviation of NN intervals (SDNN); (c) root mean square of successive differences (rMSSD); (d) percentage of adjacent NN intervals differing by > 50 ms (pNN50) (; (e) very low frequency power (VLF); (f) low frequency power (LF); (f) high frequency power (HF); (g) LF/HF ratio; and (h) estimated respiratory rate (RP).

The mixed‐design ANOVA revealed a significant main effect of time on heart rate, *F*(2, 132) = 9.76, *p* < 0.001, *ηp^2^* = 0.13, indicating changes in HR across the assessment points. In contrast, neither the main effect of group, *F*(1, 66) = 0.30, *p* = 0.586, nor the time × group interaction, *F*(2, 132) = 0.67, *p* = 0.515, reached significance, suggesting that both interventions produced comparable heart rate trajectories over time. Inspection of the mean values suggested changes in heart rate across assessment points, with a tendency toward higher values at the follow‐up assessment and comparable temporal patterns observed in both groups (see Figure 4a).

### 3.5 SDNN

The mixed‐design ANOVA revealed no significant effects for SDNN. Neither the main effect of time, F(2, 132) = 0.14, p = .867, nor the main effect of group, F(1, 66) = 0.02, p = 0.883, reached significance. The time × group interaction was also not significant, F(2, 132) = 0.64, p = 0.527, indicating that SDNN did not change across assessments and that the two interventions produced comparable patterns over time (see Figure 4b).

### 3.6 rMSSD

The mixed‐design ANOVA revealed no significant effects for rMSSD. Neither the main effect of time, F(2, 132) = 0.29, p = 0.750, nor the main effect of group, F(1, 66) = 0.72, p = 0.398, was significant. The time × group interaction also did not reach significance, F(2, 132) = 1.06, p = 0.348, indicating that vagally mediated HRV remained stable across assessments in both groups (see Figure 4c).

### 3.7 pNN50

The mixed‐design ANOVA revealed no significant effects for pNN50. Neither the main effect of time, F(2, 132) = 1.29, p = 0.279, nor the main effect of group, F(1, 66) = 0.09, p = 0.765, reached significance. The time × group interaction was also non‐significant, F(2, 132) = 0.79, p = 0.455, indicating that pNN50 remained stable across all assessment points in both intervention groups (see Figure 4d).

### 3.8 VLF

The mixed‐design ANOVA revealed a significant time × group interaction for VLF power, F(2, 132) = 3.36, p = 0.038, *ηp^2^* = 0.05, indicating that the two interventions produced different temporal trajectories. Neither the main effect of time nor the main effect of group reached significance (see Figure 4e). Follow‐up paired‐sample comparisons were conducted in accordance with the significant interaction. In the BWM‐T group, VLF decreased significantly from T0–T1, t(33) = 2.38, p = .023, but this effect was not maintained at follow‐up (T0–T2: t(33) = 0.75, p = 0.461). No significant differences emerged between T1 and T2. In contrast, the brief mindfulness induction group showed no significant changes across any time points (all p > 0.13). Overall, these findings suggest a short‐lived reduction in VLF power following BWM‐T, which was not observed after brief mindfulness induction. Given the debated physiological interpretation of VLF, this effect may reflect a transient modification in slow autonomic oscillations immediately after the intervention (see Table 4).

**TABLE 4:** Paired‐sample *t*‐tests for VLF in the BWM‐T and brief mindfulness induction groups.

Outcome	Group	Comparison	*t*(33)	*p*
VLF	BWM‐T	T0 – T1	2.38	0.023
VLF	BWM‐T	T0 – T2	0.75	0.461
VLF	BWM‐T	T1 – T2	−1.39	0.174
VLF	Brief mindfulness induction	T0 – T1	−1.20	0.238
VLF	Brief mindfulness induction	T0 – T2	0.61	0.547
VLF	Brief mindfulness induction	T1 – T2	1.55	0.130

*Note*: Paired‐sample t‐tests comparing pre‐session (T0), post‐session (T1), and 4‐h follow‐up (T2) scores on (VLF) in the BWM‐T and brief mindfulness induction groups.

### 3.9 LF

The mixed‐design ANOVA revealed no significant effects for LF power. Neither the main effect of time, F(2, 132) = 0.34, p = 0.693, nor the main effect of group, F(1, 66) = 1.01, p = 0.319, reached significance. The time × group interaction was also non‐significant, F(2, 132) = 0.10, p = 0.890, indicating that LF power remained stable across all assessments in both groups (see Figure 4f).

### 3.10 HF

The mixed‐design ANOVA revealed no significant effects for HF power. Neither the main effect of time, F(2, 132) = 0.34, p = 0.693, nor the main effect of group, F(1, 66) = 0.54, p = 0.466, was significant. The time × group interaction was also not significant, F(2, 132) = 0.10, p = 0.890, indicating that vagally mediated high‐frequency activity remained stable across the three assessments in both groups (see Figure 4g).

### 3.11 LF/HF Ratio

The mixed‐design ANOVA revealed no significant effects for the LF/HF ratio.

The main effect of time was not significant, F(2, 132) = 0.15, p = 0.861, and neither was the main effect of group, F(1, 66) = 0.02, p = 0.880. The time × group interaction also did not reach significance, F(2, 132) = 0.09, p = 0.912 (see Figure 4h), indicating that LF/HF ratio remained stable across all measurement points in both interventions (see Figure 4h).

### 3.12 RP

The mixed‐design ANOVA revealed no significant effects for estimated RP. Neither the main effect of time, *F*(2, 132) = 1.72, *p* = 0.184, nor the main effect of group, *F*(1, 66) = 0.94, *p* = 0.337, reached significance. The time × group interaction was also non‐significant, *F*(2, 132) = 0.48, *p* = 0.618, indicating that RP remained stable across all assessment points and did not differ between the BWM‐T and brief mindfulness induction groups (see Figure 4i).

## 4 Discussion

The present study examined the immediate and short‐term effects of a single session of BWM‐T and a brief mindfulness induction on psychological and autonomic indicators of stress regulation in university students. Across the two interventions, participants reported significant reductions in state anxiety and psychological distress, as well as an increase in general self‐efficacy, with changes maintained up to 4 h after the session. These findings indicate that brief mind–body practices are associated with short‐term improvements in perceived efficacy and stress‐related appraisals, consistent with prior evidence that contemplative practices can modulate attentional control and emotional arousal. (Hölzel et al. 2011; Tang et al. 2015). Importantly, given the absence of significant time × group interactions for psychological outcomes, these improvements cannot be attributed specifically to BWM‐T or brief mindfulness induction, but may reflect shared non‐specific factors such as expectancy, contextual influences, or temporal effects. Although several effects reached statistical significance, the magnitude of the observed effect sizes (*ηp^2^* and Cohen's d) was generally small to moderate. These modest effect sizes suggest that the practical significance of the findings should be interpreted cautiously, particularly in the absence of differential group effects. Ultra‐brief interventions may produce measurable short‐term shifts, but their incremental clinical impact remains to be established.

From a theoretical perspective, the convergence of effects on psychological outcomes across both interventions may reflect shared regulatory mechanisms. Although BWM‐T and brief mindfulness induction may rely on partially distinct bottom‐up and top‐down mechanisms, their shared effects on attention and interoception may explain the similar short‐term outcomes observed. While theoretical distinctions between bottom‐up and top‐down regulatory pathways remain conceptually meaningful, the largely overlapping trajectories observed across groups indicate that these mechanisms did not translate into clearly differentiated short‐term outcomes in the present design.

Models of stress appraisal and self‐regulation suggest that decreases in sympathetic arousal and perceived threat can quickly enhance the sense of control and efficacy (Bandura 1997; Lazarus et al. 1984). The increase in self‐efficacy observed at the four‐hour follow‐up may therefore represent a downstream effect of the acute reductions in anxiety and distress, rather than a direct effect of the intervention techniques themselves.

In contrast to the psychological outcomes, autonomic indices demonstrated a more selective pattern of change. A significant group × time interaction emerged only for VLF power, with a transient reduction immediately after BWM‐T that was not observed in the brief mindfulness induction group. Although the physiological interpretation of VLF remains debated, especially in short‐term recordings, reductions in this band have been tentatively associated with changes in slow homeostatic processes, thermoregulation and long‐latency autonomic oscillations (Shaffer et al. 2017).

Importantly, heart rate showed a significant main effect of time, indicating temporal modulation of cardiac activity across assessments, but without group differences or divergent trajectories. In contrast, estimated RP remained stable over time and did not differ between groups. This dissociation suggests that the observed autonomic changes cannot be attributed to simple alterations in breathing frequency, but rather reflect more complex cardiorespiratory dynamics. In line with contemporary psychophysiological frameworks, this pattern supports an interpretation of HRV and cardiac changes as emergent properties of integrated regulatory processes, rather than direct proxies of isolated vagal modulation (Quigley et al. 2024).

Although it is theoretically plausible that BWM‐T may engage bottom‐up regulatory pathways, the present data do not allow strong conclusions regarding mechanism‐specific effects, given the largely overlapping group trajectories. A more detailed interpretation is required for the VLF findings, especially given that this was the only HRV parameter showing a significant interaction, but emerging evidence suggests that mind–body and contemplative interventions can transiently modulate VLF, even when other HRV indices remain unchanged (Tang et al. 2015; Bernardi et al. 2006).

An important contribution of this study lies in the adoption of a 4 h follow‐up window. Most research on single‐session interventions examines outcomes immediately after practice or within one to 2 h. Extending the window to four hours addressed a meaningful ecological question, whether rapid psychological improvements persist during the resumption of daily academic demands, when stressors naturally re‐emerge. The maintenance of reductions in anxiety and distress at T2 suggests that brief mind–body practices may offer functional short‐term support during periods of academic pressure, even if physiological changes remain circumscribed or difficult to detect. Importantly, this reduction was not maintained at the 4 h follow‐up (T2), which substantially limits its theoretical and practical relevance. The absence of persistence suggests that this effect may reflect a short‐lived physiological fluctuation rather than a stable regulatory shift.

Overall, these findings provide further support for the short‐term psychological benefits of brief mind–body interventions, while highlighting the difficulty of detecting parallel autonomic changes.

Differences between bottom‐up and top‐down techniques may emerge primarily in their immediate embodied signatures, such as the transient VLF reduction following BWM‐T, whereas subjective emotional improvements appear to be shared across modalities. Further research using larger samples, more fine‐grained autonomic measures, and ecologically valid stress manipulations is needed to clarify the specific pathways through which these interventions exert their influence.

### 4.1 Limitations and Future Directions

A first limitation concerns the absence of a passive or sham control condition. Although the comparison between two active interventions (BWM‐T and brief mindfulness induction) is methodologically appropriate for evaluating relative effects, the present design does not allow conclusions regarding absolute efficacy compared to no intervention or simple rest. Because both groups received structured and time‐coordinated interventions and showed comparable improvements over time, it remains unclear whether the observed changes in anxiety, distress, and self‐efficacy reflect intervention‐specific mechanisms, non‐specific factors such as attention, expectancy, or demand characteristics, simple time or rest effects, or regression to the mean. This limitation is consistent with broader methodological challenges in psychotherapy research, where the construction of adequate placebo or sham conditions is inherently complex due to the influence of common factors and the difficulty of double‐blinding (Locher et al. 2018, Kim et al. 2019). Active control conditions may reduce overestimation of treatment effects, yet they also make it more difficult to isolate intervention‐specific mechanisms, as shared therapeutic elements (e.g., credibility, expectancy, relational factors) can contribute substantially to outcomes.

This issue is particularly relevant for psychological outcomes, which are known to be sensitive to contextual and expectancy influences, but also for HRV measures, which may vary due to posture, quiet sitting, or spontaneous relaxation. In the absence of a passive or attention‐matched control condition, physiological changes, especially the isolated VLF finding, cannot be clearly disentangled from non‐specific relaxation or time‐related influences. The present study was therefore designed to address comparative short‐term effects between two active regulatory practices rather than to establish efficacy against no intervention. Future research including passive, waitlist, or structurally equivalent attention‐matched sham conditions will be necessary to better isolate incremental and mechanism‐specific effects of BWM‐T.

A second limitation concerns the fact that despite an a priori power analysis was conducted, the sample size provided adequate sensitivity only for medium‐sized interaction effects. Smaller physiological effects, particularly those affecting HRV indices, may therefore have remained undetected. HRV parameters are characterized by substantial inter‐individual variability, and detecting subtle changes following ultra‐brief interventions typically requires larger samples or repeated‐measures intensive designs. Therefore, the absence of significant time × group interactions for most outcomes should not be interpreted as evidence of true equivalence between interventions but rather as potentially reflecting limited statistical power to detect small interaction effects. Although the a priori power analysis was conducted assuming a medium effect size (*f* = 0.25), this estimate may be considered optimistic for ultra‐brief interventions and for physiological outcomes such as HRV, which often exhibit small and highly variable effects. Given the number of psychological and physiological outcomes examined, the possibility of Type I error cannot be excluded, particularly for isolated significant findings.

A third limitation, several known covariates of HRV were not experimentally controlled or statistically adjusted for, including recent physical activity, caffeine intake, sleep quality, menstrual cycle phase, and respiratory patterns. Participants were instructed to avoid substances and activities that could influence autonomic activity, but compliance was not objectively monitored, which may have introduced uncontrolled variability into the HRV measures, despite the standardized behavioral restrictions implemented prior to assessments. Breathing was not externally paced or directly measured via respiratory sensors, which may limit precise interpretation of frequency‐domain HRV indices.

A fourth limitation is that psychological measures relied on self‐report instruments, which are subject to expectancy effects, response biases, and limited temporal resolution. In particular, the use of a single‐item distress scale and a GSES measure may not fully capture rapid fluctuations in stress and regulatory processes within a four‐hour timeframe. This may have attenuated the sensitivity to detect subtle short‐term changes.

Fifth limitation, the choice of a 4 h follow‐up, although theoretically grounded and ecologically meaningful, still represents a relatively short observational window. It remains unclear whether the observed psychological benefits would persist over longer intervals or across repeated exposures to academic or environmental stressors. Future research should therefore incorporate additional follow‐up assessments, objective markers of stress reactivity, and designs allowing for direct comparison with control conditions.

Although HF and SDNN did not reach statistical significance, both indices showed trends that may be physiologically meaningful. To better interpret these patterns, future research should include a passive control group, which would allow determination of whether such tendencies reflect genuine autonomic responses to the interventions rather than spontaneous variability.

## 5 Conclusions

In conclusion, the present study suggests that a single 16 min session of BWM‐T or a brief mindfulness induction was associated with rapid improvements in self‐reported anxiety, distress, and perceived self‐efficacy in university students, with benefits persisting for at least 4 h. These findings support the potential value of ultra‐brief mind–body practices as accessible tools for short‐term regulation in academic contexts. However, given the non‐clinical nature of the sample and the limited 4 h follow‐up, these results should not be generalized to clinical populations or interpreted as evidence of sustained therapeutic impact beyond acute, short‐term effects. Autonomic responses, however, were limited, aside from a transient modulation of VLF following BWM‐T, no significant changes emerged across other HRV parameters. This pattern suggests that subjective improvements may precede or exceed detectable shifts in cardiac autonomic activity when interventions are delivered in such a brief format. Future research employing larger samples, longer follow‐up periods, objective markers of stress reactivity and appropriately controlled designs is needed to clarify the specific psychophysiological mechanisms through which brief mind–body interventions exert their effects and to determine their practical utility in real‐world academic environments.

## Author Contributions

**Marco Borgese**: conceptualization, investigation, writing – original draft, methodology, validation, visualization, writing – review and editing, software, formal analysis, data curation, resources.

## Funding

The authors have nothing to report.

## Ethics Statement

The study was conducted in accordance with the Declaration of Helsinki and approved by the Ethics Committee of the University of Salerno (reference number: 0186309, approval date: June 11, 2024). All participants provided written informed consent prior to participation.

## Conflicts of Interest

The authors declare no conflicts of interest.

## Data Availability

The data supporting the findings of this study are available from the corresponding author upon reasonable request.
